# An interdisciplinary approach to the Loss and Damage fund: exploring potential applications and guiding principles

**DOI:** 10.14324/111.444/ucloe.3042

**Published:** 2026-04-23

**Authors:** Penlope Yaguma, Simon Chin-Yee, Priti Parikh, Lisa Vanhala, Mark Maslin, Richard G. Taylor, Mariam Zaqout, Carole Roberts, Jacqueline McGlade

**Affiliations:** 1 Department of Science, Technology, Engineering and Public Policy, University College London, UK; 2 Department of Political Science, School of Public Policy, University College London, UK; 3 Engineering for International Development Centre, Bartlett School of Sustainable Construction, University College London, UK; 4 Department of Geography, University College London, UK; 5 Institute for Global Prosperity, University College London, UK

**Keywords:** Loss and Damage fund, climate change, climate justice, water resources, infrastructure, climate finance

## Abstract

After decades-long advocacy by low- and middle-income countries, the establishment of a Loss and Damage fund during the Conference of Parties 27 was monumental. The Loss and Damage fund unlocked practical avenues to climate justice. It recognises the responsibilities of rich countries with respect to greenhouse gases that exacerbate climate change and disproportionately impacts vulnerable countries. With the fund still in its infancy, it is crucial to differentiate between types of finance that are suitable for other forms of climate action from finance intended specifically to address loss and damage. We consider potential applications of the funding for different sustainable development issues including water resources, energy, transport, human rights and security for a high-functioning Loss and Damage fund. We also highlight the need for the Loss and Damage finance to be attentive to and be able to redress some of the root causes of vulnerability while providing low- and middle-income economies with the support they need in the face of climatic impacts. We propose four guiding principles towards a high-functioning Loss and Damage fund: consistent contributions, clarity in governance, community-driven solutions and fund allocation, and corruption-free systems. We argue that applying those principles promotes climate justice and human rights, and inclusive fund governance. Being community-driven requires such funding to be reactive to climate events, yet proactive in understanding affected communities’ needs so that finance allocated is not just a band-aid solution but a long-term and just solution to climate resilience.

## Introduction

In 2015, when the Paris Agreement was adopted during Conference of Parties (COP) 21, it included a separate article on Loss and Damage (L&D) establishing the topic as the third pillar of climate change policy; distinct from mitigation and adaptation. Although the interlinkages between the three pillars are strong, with overlaps in activities as highlighted by the work of the United Nations Framework Convention on Climate Change (UNFCCC)’s adaptation committee, averting, minimising and addressing losses and damages from climate change will require protective measures (adaptation) and a reduction in greenhouse gas emissions (mitigation) as well as distinctive reparative measures [1]. Over the course of their discussions in 2023, the UNFCCC Transitional Committee tasked with establishing a new fund and financial arrangements to address L&D reached consensus on some matters but key issues remain unresolved. For instance, the current project-based climate finance often undermines the potential to integrate with wider national funding and planning processes in the recipient country [2]. By taking an interdisciplinary approach, we demonstrate the merits of creating a fund distinctive from existing financial mechanisms for adaptation or mitigation measures. This section presents the key facets of both the science and the global governance of L&D followed by an interdisciplinary analysis of how L&D specific funding could be deployed. Finally, we lay out a set of principles that could guide the operationalisation of the L&D fund.

### Defining loss and damage

While the term ‘loss and damage’ does not have an official agreed upon definition, highlighting the contentiousness of this area of the climate change negotiations, a 2012 UNFCCC literature review defined L&D as ‘*the actual and/or potential manifestation of impacts associated with climate change in developing countries that negatively affect human and natural systems*’ [3]. For many, ‘loss and damage’ refers to those negative climate impacts that we as an international community have not prevented (or have not chosen to prevent) through mitigation or accommodated through adaptation measures. Many scholars use the term L&D to refer to the political negotiations under the UNFCCC and the wider policy agenda on the topic of residual climate change impacts [4], whereas the lower case ‘losses and damages’ deployed in the Intergovernmental Panel on Climate Change (IPCC) Sixth Assessment Report is used to refer to harm from (observed) impacts and (projected) risks from climate change [5]. We make this distinction throughout this paper.

Scholars and policymakers use different frameworks for understanding what L&D is, and the relationship between this area of policy making and adaptation. For example, Mechler et al. [6] highlight the distinction among avoided, un-avoided and unavoidable losses and damages. They suggest that avoided losses and damages can and will be averted or minimised through mitigation efforts, adaptation interventions and effective disaster risk reduction (DRR) techniques (e.g., changing crop varieties to accommodate increasing temperatures or planting mangroves to slow down coastal erosion). Un-avoided losses and damages are risks that have not or could not have been avoided due to resource or capacity constraints, but where there was, at some stage, the possibility of doing things differently to avoid loss. Unavoidable losses and damages are risks and impacts that exceed the capabilities of existing mitigation and adaptation measures; hence they have irreversible impacts, such as glacier melt. Quite often those are irreversible, such as permanent damage to crop cycles and non-economic losses in society affecting social fabric [7]. Mechler et al.’s [6] framework is useful for highlighting both the decision-making and temporal dimensions of various forms of climate action.

Losses and damages can result from slow-onset events, such as increasing temperatures, desertification, the degradation of land and forests, retreating glaciers and sea level rise or from climate change-associated extreme weather such as cyclones, floods, heatwaves, droughts, wildfires and storm surges [8]. While analytically useful, the problem with dichotomising climate change drivers of L&D in this way is that it ignores the compounding and cascading nature of climate change impacts. For example, a coastal city that faces storm surges is put at greater risk both through the rising of sea levels and the increased frequency and intensity of storms that produce those surges. The latest IPCC report has helped to focus policymakers, attention on this issue of compounding and cascading climate risks [9].

Another dichotomous categorisation often talked about in the context of loss and damage is the distinction between economic losses, such as impacts on business productivity, damage to infrastructure and buildings and declining agricultural productivity, and what has been referred to as non-economic losses, which are those losses or damages that might be more difficult to monetise, including loss of life, negative impacts on physical and mental health, a degrading of social cohesion and impacts on human mobility [10]. Within this is the loss of cultural heritage, both tangible in terms of loss of land and infrastructure important for a community, but also intangible, by taking away cultural practices like the disappearance of glaciers representing deities at the centre of cultural cosmologies in East Africa (Uganda and the Democratic Republic of Congo) and the Andes, or the loss of spices/foods needed for important culinary traditions [11,12]. Negative environmental consequences such as biodiversity loss and the degradation of ecosystem services have also been grouped under this heading.

### How is the L&D fund different from other funds?

While newspaper headlines celebrated the establishment of an L&D fund in the wake of COP27, it is critical to note that the text outlining the decision referred to ‘new funding arrangements’ including ‘a fund for responding to loss and damage’. Behind this language is a distinct divide between high-, low- and middle-income countries over whether a new body should be set up or whether existing funding arrangements should be reformed. The insufficiency of finance for dealing with loss and damage is seen as a major issue for vulnerable countries who are bearing the brunt of the impacts [4]. They have been advocating for new and additional finance in this area for more than a decade, but the pace of progress has been very slow. When understood in the context of the failure of the high-income countries to deliver the 100 billion USD per year for mitigation and adaptation measures that they promised at the conclusion of the Copenhagen negotiations at COP15 in 2009 [13], there is a degree of frustration and a sense of urgency among low-income countries to establish a new fund. They specifically have been calling for a fund that is well-suited to the specific challenges of addressing L&D: they see this as moving away from a project-based, loan-focused way of providing finance and towards the provision of grants that can be rapidly disbursed after climate change impacts have hit. Because the UNFCCC already runs four different funds to address climate change, which have had varying degrees of success in terms of effectiveness and legitimacy it is important to understand how best to design a fund that can deal with the needs of low- and middle-income economies.

Insurance has often been posited as one way of addressing L&D (it is worth noting that the term ‘loss and damage’ itself emanates from the sphere of insurance), but a now voluminous body of academic and grey literature highlights the inadequacy of insurance alone as a financial mechanism that can help countries grapple with loss and damage [14]. Ongoing discussions among the Transitional Committee, have sought to understand and identify relevant sources of finance outside the UNFCCC. These lie mainly in the realm of humanitarian assistance and development aid. Some argue that humanitarian funding, which tends to be reactive and discretionary, is unsuitable, not only because levels of such finance are insufficient to meet needs in the wake of disasters but often such funding is only triggered when a disaster reaches a certain scale [15]. While new initiatives with forecast-based financing and early action are being put in place they are not yet at a scale able to address the magnitude of the losses and damages already being experienced [16]. There are also finance gaps in terms of rehabilitation and reconstruction after climate-driven weather events. In terms of development funding, Small Island Development States (SIDS) and Least Developed Countries have highlighted some of the negative spirals associated with disasters and debt, showing that the use of concessional finance can overburden vulnerable countries with debt following a disaster: the combination of increased spending and reduced revenue threatening national fiscal sustainability. In 2016, the Forum of the Standing Committee on Finance recognised a lack of finance to address slow-onset events, meaning that many countries are getting left behind in the adaptation space, rendering them more vulnerable to loss and damage. Pill [17] interviewed 43 global L&D practitioners, who noted that the concepts of DRR, preparedness, adaptation, resilience and avert/minimise, showed the largest agreement for inclusion in L&D. This is particularly pertinent for investment in infrastructure as it enables funding to be channelled to rebuilding a climate-resilient infrastructure [18].

Although the concept of an L&D fund could meet unaddressed needs in the international community, particularly for countries and communities that are amongst the most vulnerable, its practicable implementation requires a recognition of the many social, economic and environmental factors that constitute losses and damages. In this study we set out a series of examples of how some of these factors interrelate and from that derive some emergent principles that might be adopted to help make a high-level L&D fund succeed in improving resilience to climate change across a broad range of types of need. Our study cannot be considered comprehensive; but it may be that the principles could be applied beyond the areas we have drawn on as examples of climate impacts causing loss and damage. The suitability of these principles could be tested in further academic studies or even real case studies employing them.

### Loss and damage and attribution

The way L&D is defined shapes our perceptions on who can access the fund and for what. To do so, it is crucial to understand the impact of both human activity and extreme weather events on the most vulnerable countries. The IPCC Sixth Assessment Report [5] states that it is virtually certain that anthropogenic climate change has caused increases in the frequency and severity of hot extremes and decreases in cold extremes on most continents. The frequency and intensity of heat waves have increased around the world with record-breaking heat waves over the last decade. Global warming is also the main cause of the observed intensification of precipitation, resulting in fewer but heavier precipitation events and the exacerbation of flooding. Record-breaking extreme floods have, for example, been recorded over the past decade in Brazil, Great Britain, Canada, Chile, China, East Africa, Europe, India, Indonesia, Japan, Kenya, Korea, Mozambique, the Middle East, Mozambique, Niger, Nigeria, Pakistan, South Africa, Thailand, Uganda, USA and Vietnam [19]. Most of those nations are amongst the most vulnerable geographies to climate loss and damage.

**Figure 1:**
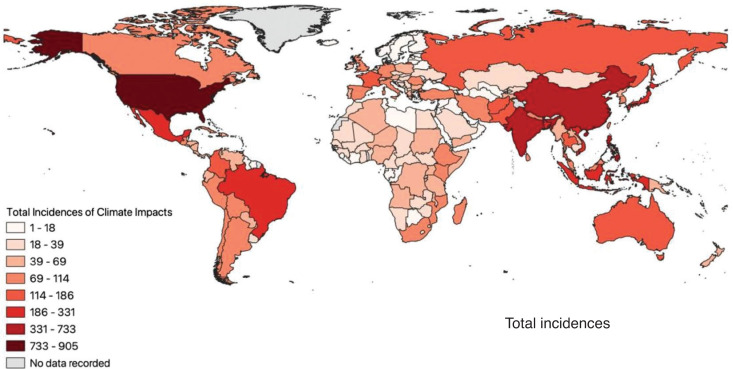
Total climate-related impacts per country between 1980 and 2022. Source: Compiled with data from The Emergency Events Database (EM-DAT) (Centre for Research on the Epidemiology of Disasters – Université catholique de Louvain, [23]).

One reason why scientists have growing confidence that many of these extreme weather events are exacerbated by climate change is advancements in attribution science [20]. Attribution science is a field of research that connects climate events or trends to human-caused climate change [21]. Improved computer processing power and methods for modelling the factors that contribute to weather allow scientists to run weather simulations for a region with and without the influence of anthropogenic greenhouse gases. These procedures have been done for decades, but more recently, the sophistication and statistical robustness of the methods have drastically improved. This allows us to determine the probability/likelihood by which climate change has contributed to individual extreme weather events and whether it has increased the intensity or the frequency or both. Over 113 extreme weather events that occurred between 2015 and 2020 have been studied using attribution science [22]. 70% of events were found to have increased frequency or intensity due to climate change, 26% were found to have a reduced occurrence due to climate change and 4% showed no variation due to climate change. Figure 1 illustrates the global distribution of climate-related impacts between 1980 and 2022. The figure shows that countries and communities that are most vulnerable bear the burden of climate change impacts.

## Cross-sectoral benefits of a high-functioning L&D fund

**Figure 2:**
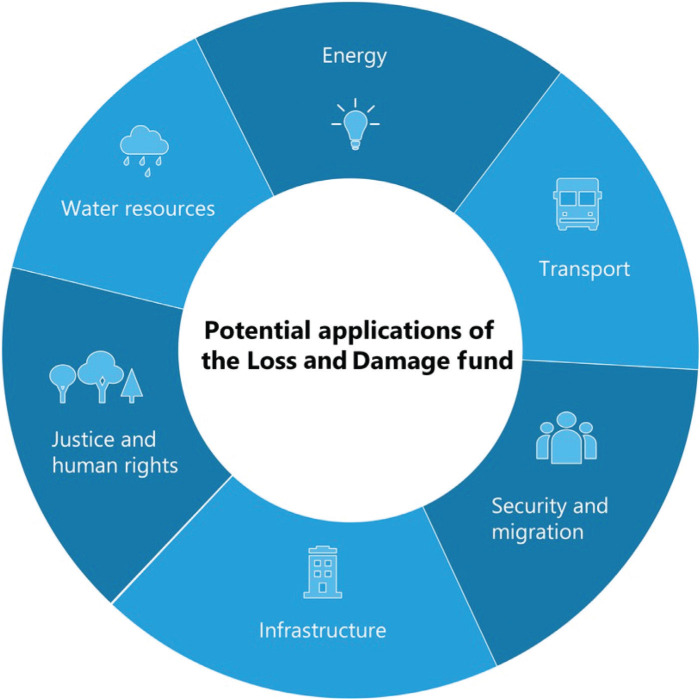
The potential applications of the L&D fund across different sustainable development issues.

Estimates show that the number of climate migrants/refugees could rise to 1.2 billion people by 2050 [24]. Migration overall leads to populations living in a humanitarian crisis deprived of access to basic infrastructural services. The L&D fund could address infrastructure service delivery for migrants/refugees, who often move into locations with inadequate provision of infrastructure. This moves into the debate of whether funds – for adaptation or L&D – could be used for longer-term development needs and addressing existing inequities in infrastructure services. In this section, we consider how L&D funding could be deployed within critical sustainable development challenges that are central to the wellbeing of climate migrants but also the wider global population. We refer to ongoing or recent events, highlighting practical problems and gaps that the fund could address in water resource management, sanitation, energy and transport, human rights and justice as summarised in Fig. 2. We examine these different challenges across sectors to showcase the multifaceted impact of climate change on sustainable development in the most vulnerable countries.

### Water resources management and sanitation infrastructure

The hydrosphere is the world’s largest distributor of heat [25]. Thus, the oceans play a major role in climate regulation and as a consequence of this is there are changes within the hydrological cycle that affect rainfall and other aspects of both the hydro- and cryosphere. All this can affect the availability of freshwater for human use and biodiversity. The latest assessment report of the IPCC [9] reviews the evidence of these consequences, which include precipitation and temperature extremes exacerbating the risk of floods, drought and wildfires in desiccated landscapes. The magnitude of these changes observed that just over 1 °C of global warming over the last half century has been greatest in the tropics, affecting several low-income counties [26]. Therefore, addressing the consequences of global warming in these areas is a matter of climate justice as pollution produced by high-income countries during industrialisation and the continuous use of non-renewable energy to drive economic growth both in-country and abroad are key contributors to global warming. The IPCC report also highlights the impacts of sea level rise and increased risks posed by storm surges as well as the decline in freshwater stored in seasonal snowpacks, alpine glaciers and continental ice sheets.

For water resources, a high-functioning L&D fund could address a myriad of consequences and vulnerabilities affecting stakeholders in a range of environments. One pervasive consequence of the intensification of precipitation (i.e., fewer but heavier precipitation events) in a warming world is a reduction in soil moisture at certain times. This impact, combined with changes in the seasonality and predictability of precipitation, is projected to increase freshwater demand for irrigation. Such increases are expected to be most pronounced in low-income countries of tropical Africa seeking to regularise and reduce their vulnerability of food production that currently depends on rain-fed agriculture [27] as well as Eastern Mediterranean and Middle East water resources and agriculture [28]. Indeed, the damage to food production and livelihoods from the recently observed decline (loss) of the ‘long rains’ (March–April–May rainy season) in the Horn of Africa is considerable [27]. L&D funds could, for example, address loss and the consequences of damage through community-led solutions (e.g., seed banks, changing crop types, small-scale irrigation) as well as improved seasonal forecasts, and the delivery of climate information.

Other permanent consequences of climate change include the loss and reduction of tropical alpine glaciers in the East African Highlands (e.g., Kilimanjaro, Mount Kenya, Rwenzori Mountains) and the Andes [29]. Deglaciation affects not only distinctive alpine ecosystems that are hotspots of biodiversity but also water supplies (e.g., Lima, Peru) and cultural references as the cosmology (worldview) of communities living in these regions often revolves around the presence of glaciers. How L&D funds can be used to address the injustice of climate change impacts experienced by communities with little or no responsibility for these requires a longer conversation analogous to discussions taking place with indigenous communities around the world (e.g., Canada, USA, New Zealand) regarding the historical theft of lands [30]. Colonisation and centuries of resource exploitation are at the very heart of climate injustice and continues to affect action on climate change in low-income countries, exacerbating global inequalities [31]. For instance, in low-lying regions such as the Bengal Basin of Bangladesh and in SIDS such as the Maldives, global sea level rise together with the salinisation of coastal water supplies is leading to forced migration. L&D funds could be used to compensate those who have lost their land, homes and livelihoods and offer sustainable and just alternatives.

An increased frequency and intensity of flood events is a key consequence of climate change. During the COP27 period, the International Federation of Red Cross (IFRC) responded to 74 floods globally [32]. Floods in Pakistan caused US$30 billion in damages, which is beyond what national governments can fund [32]. L&D funds have the potential to leverage investment towards floods resilience and sustainable water resources management through local, regional and national water resource management (e.g., rainwater harvesting and nature-based solutions) and delivery of water infrastructure.

With sanitation infrastructure, it is likely that the global efforts to meet Sustainable Development Goal 6, particularly Target 2, which ensures universal access to adequate and equitable sanitation and hygiene by 2030, will not be enough as more than 3.4 billion people still lack access to safe sanitation services [33]. The sector employs on-grid and off-grid (on-site) sanitation solutions to address current service gaps. Most off-grid sanitation options such as septic tanks and pit latrines are at risk of increased flooding [34]. Such off-grid solutions are mostly deployed in low-income settings, such as slums, which are already more vulnerable to climate shocks and stresses. In several countries, including Bangladesh, South Africa and Kenya, such infrastructure was part of the colonial urban systems that separated the coloniser from the indigenous population [35]. Thus, there is a fundamental need to draw connection between achieving universal access to sanitation (regardless of the type of solution) and the role of an L&D fund. This is particularly relevant in post-climate disaster response to address public health and economic productivity [36].

### Energy and transport

Climate impacts such as sea level rise, heavy rains and storm surges, and extreme heat impact transport. These risks weaken or damage roads, bridges, railway lines, airports and ports, compromising the performance, safety and reliability of transport systems. Mudslides and landslides block transport networks, sometimes cutting off entire communities or regions [37]. The Third UK Climate Change Risk Assessment found that an increase in the number and intensity of heatwaves caused road surfaces to soften, train tracks to buckle and signalling failures as has been observed during the most recent summers [38]. The key challenge with rebuilding climate-resilient transport infrastructure is the disconnect between the longer timelines of delivering large-scale public transport and the short- to medium-term financial constraints that governments often face. L&D funds could play a role in plugging this short- to medium-term resource gap.

In July 2023, the International Maritime Organization agreed its revised Greenhouse Gas Strategy. This non-legally binding agreement is a set of guiding principles on decarbonisation rates and a ‘broad’ timeframe in which this should be achieved. While this agreed strategy has been criticised for not aligning with the Paris Agreement’s 1.5 °C temperature goal (it is not as ambitious as the IPCC AR6 report stipulates), it was a step forward for the global shipping community in agreeing to the need to decarbonise their sector [39]. Shipping is a critical mode of transport for people, goods, energy and communities all over the world, but especially for island states that are remote and reliant on goods being shipped in. For example, the Comoros, despite being an agricultural country, imports over US$100 million of food products annually [40], and the Marshall Islands rely on shipping for their energy needs. By taking the shipping sector into account, an L&D fund has the potential to provide guidance on how countries can invest in both using this sector for their economic and social needs while at the same time greening the sector itself to become more sustainable. Additionally, the fund is an opportunity to engage the sector when a natural disaster occurs, both in getting aid/goods to those in need in the aftermath of an extreme weather event, while at the same time building the sector back better to avoid future losses and damages. Largely ignored in the UNFCCC space, the maritime sector could play a much bigger role in addressing the economic realities of vulnerable or remote countries.

Post disaster, the L&D fund likewise would create incentives to invest in resilient energy systems. Large, centralised energy systems such as hydropower dams affected by prolonged droughts and flooding events can be replaced with off-grid and decentralised energy solutions that diversify the energy mix and boost climate resilience. Similarly, the L&D funds can support and incentivise communities to transition to cleaner cooking fuels such as biogas and electricity, which would have the twofold effect of being healthier for users as well as limiting wildfires as a result of biomass. Whilst it would be difficult to make the case for using L&D funds to subsidise clean fuels in their entirety, this could be used as a principle for building new energy systems, or the replacement of clean fuels in the transport sector when rebuilding infrastructure such as ports or ships. Given that one of the key objectives of the L&D fund is to boost resilience, this would only be achieved in a setting where existing water, sanitation and energy infrastructure meet current needs, and the funds could be used to top up following a crisis.

### Human rights

Climate change has been referred to as a significant human rights challenge in this century, as its losses and damages pose a severe threat to the rights of affected communities [41]. This includes fundamental rights, such as the right to life; and economic, social and cultural rights, such as the right to work, education and the highest attainable standard of physical and mental health, and adequate food and housing [42]. Loss and damage impacts also compound more systemic risks that could exacerbate threats to civil and political rights as well, such as liberty and property rights. While a general survey of existing and potential future human rights violations driven by L&D is beyond the scope of the paper, it may be worth highlighting that literature in disaster studies shows that human rights violations increase during climatic and weather-related disasters. For example, violence against women and girls substantially increases and the consequence of the mass movement of people fleeing extreme weather events results in human rights violations [43].

By adopting a human rights-based approach, the fund could take a strategic approach in strengthening the UNFCCC’s response to L&D. Toussaint and Martínez Blanco argue for the integration of the normative dimension of human rights into the design, implementation and evaluation of policies and actions on L&D and this could apply to the development of funding arrangements [44]. It is worth noting that a human rights-based approach emphasises the obligations states already have under existing international and regional human rights treaties in addition to the language on human rights included in the preamble to the Paris Agreement. Taking a human rights-based approach to providing funding for L&D would entail both substantive commitments to protection, respecting and promoting human rights in the activities that are funded, but also ensuring that the fund’s governance processes and mechanisms of fund allocations are participatory, inclusive and non-discriminatory.

### Human security

In taking a human rights-based approach to understanding both human security as well as the delivery of L&D funding, many of the injustices and pitfalls of the funds that have come before it could be avoided. For many around the world, climate change has exacerbated insecurity – natural disasters (fires, floods, cyclones) and slow-onset events (sea level rise, desertification and drought) combined with poverty levels and epidemics lead to migration and conflict, undercutting any chance for sustainable development [45]. The General Assembly resolution 66/290 has called for ‘*people-centred, comprehensive, context-specific and prevention-oriented responses that strengthen the protection and empowerment of all people*’ [46]. Arguably, any new fund on L&D needs to both be reactive to extreme weather events, but also predictive in understanding those communities (not just countries) most vulnerable in order to be prepared to react but also be able to ‘build back better’ [47]. Meaning, they must understand the complex needs of these communities so that any finance allocated is not just a band-aid solution but goes to the root causes of vulnerability.

SIDS are particularly vulnerable (Pacific, Caribbean, Indian Island delegates). Geographically, they are not only small in physical size but tend to have single commodity economies that can be devastated following an extreme weather event. In addition, many island states (Comoros, Tonga, Kiribati, etc.) have reported an increase in the frequency and intensity of cyclones and hurricanes; in the Comoros, Cyclone Kenneth in 2019 devastated the country’s burgeoning fishing industry. Although the Comoros Islands had experienced cyclones in the past, they were unprepared for the sheer strength of this storm. A well-functioning L&D fund could inject finance into the communities in the immediate post-disaster period. The 2017 Hurricane season in the Caribbean was particularly devastating. Hurricane Irma ripped through Barbuda damaging 90% of the property [48]. Only two days later, Hurricane José followed a similar path to Irma, and the entire population of the island was evacuated to nearby Antigua. Many islands in the region are overly dependent on tourism revenue, and in the wake of a natural disaster it can take years to build back damaged infrastructure, as well as attracting tourists back. Through linking climate and environmental justice to sustainable development [49], an L&D fund has the opportunity to build back equitably, where the tourism economy is balanced with the need to serve the local communities, including building roads, addressing energy needs, and health crises – see the 2010 earthquake recovery efforts in Haiti or the 2017 hurricanes that affected Barbuda and Puerto Rico among others [50]. Tonga has also been trying to push the international community to recognise a new category 6 hurricane/typhoon (current scales go to 5), as they have been experiencing very strong tropical storm winds of over 250 km/hour in recent years. In 2018, Cyclone Gita hit Tonga as they were trying to recover from Cyclone Ian that occurred four years previously (Tonga Delegate, COP27). From the beginning, it has been the Pacific Island states that have been particularly forceful in pushing for an L&D fund at the UNFCCC, as their whole identity, culture and way of life is at risk because of hurricanes and sea level rise.

Countries in Sub-Saharan Africa have also pushed for an L&D fund. Desertification and droughts, extreme weather events and even plagues of locusts have increased greatly in recent years. As countries are facing widespread climatic events, this has resulted in economic instability and loss of lives and livelihoods leading to insecurity and conflict over remaining resources [51]. In 2019, Cyclone Idai made landfall in East Africa, this was followed by Cyclone Kenneth, which also hit Comoros. The immediate storm was catastrophic to the town of Beira, and the subsequent flooding affected nearly 2.2 million people in Mozambique, Zimbabwe and Malawi. A year later, over 100,000 people were still living in resettlement sites [52]. For Mozambique, an L&D fund could address not only the immediate needs of communities suffering from destroyed infrastructure, loss of lives as well as health concerns (cholera outbreaks), but also the longer-term economic losses for towns and businesses, and the loss of agricultural productivity.

Taking a grassroots level approach to reconstruction allows alternative forms of sustainable development that are not just about building back but also provide pathways to ‘more resilient and regenerative tourism practices in tourism’ in island states [53]. It needs to be both urgent and quick moving when a community is devasted by fires, floods and cyclones, but can also be used to put proactive policies in place that can build back infrastructure that is both climate resilient (in terms of buildings, roads, etc.) and climate friendly (use of green technologies, energies, etc.) [54].

## Proposed guiding principles

This paper suggests that, at its best, an L&D fund has the potential to address structural issues in society and can help to address some of the root causes of vulnerability. However, there is also the risk that it becomes another bureaucratically cumbersome and inefficient institution that is not able to meet needs in a rapid-response and community-driven way when a community is devasted by fires, floods, cyclones, etc. There is also a risk that it fails to deliver the scale of funding that is required to meet the challenges associated with the wide range of losses countries are already facing because of climate change.

Our interdisciplinary study highlights the need for the fund to emphasise building back infrastructure that is both climate resilient (in terms of buildings, roads, etc.) and climate friendly (use of green technologies, energies, etc.). We have also shown that it is important to be aware of the social dimensions of an L&D fund which entails participation of recipient communities in decision-making, championing human rights-based approaches in post-disaster recovery while also prioritising human security. Throughout this paper we have made the case that several sustainable development issues such as access to water and sanitation, energy and transportation, and the realisation of human rights and human security would benefit if such a fund was both established and equitably run. It is through taking a climate justice approach to an L&D fund that those sustainable development issues can be addressed. Funding crucial social infrastructure such as water, energy and transport should be balanced with the processes of building back the economy after a natural disaster. For instance, economic sectors such as tourism and the food industry could address the short- and long-term needs of the society, who are most vulnerable to climate shocks, through pooling or mobilising an L&D fund towards issues such as public health and food security.

**Figure 3:**
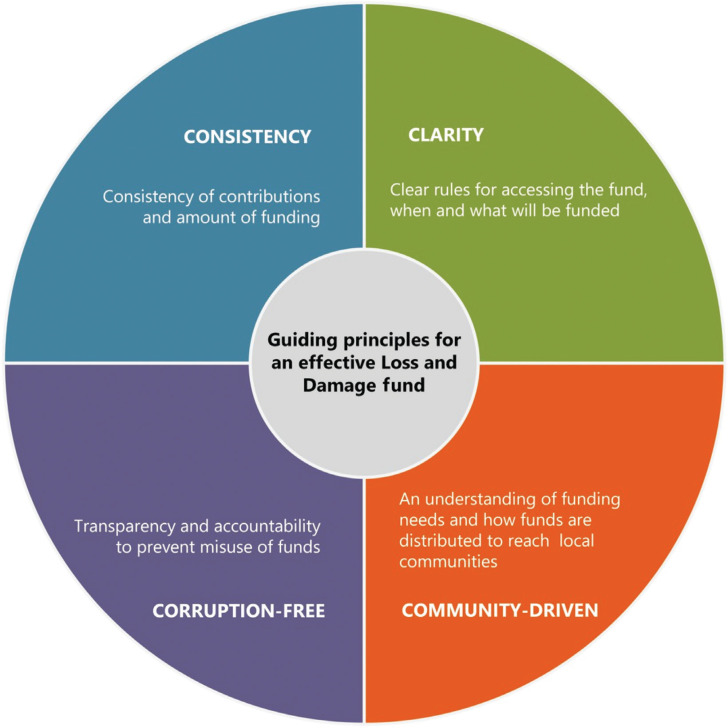
Principles that could guide an effective L&D fund.

Based on this analysis we propose guiding principles: consistency, clarity, community-driven and corruption-free, summarised in Fig. 3, that need be taken into consideration if we are to ensure effective and equitable distribution of new forms of climate finance. If developed and implemented, these principles could help overcome barriers and enhance the impact of L&D funds.

### Consistency

As the potential design of the L&D fund is still unfolding, the financial commitments of countries, especially high-income ones, are still questionable. Promises towards the L&D fund are still voluntary, which is partly why the L&D fund is still a contested proposition, and this poses the question of reliability [55]. This fund needs consistency in the amount of funding, how it is distributed and timescales of distribution. Reliable funding streams are essential for long-term planning and implementation. It is also necessary that there should be a window for rapid disbursement of funding when necessary. Unlike the experience with the Green Climate Fund, countries should commit to providing finance consistently year after year. This will provide the stability often missing when looking at L&D. If countries know that financial support will be made available, they will be able to plan proactive resilient and/or clean energy practices into their projects, while at the same time knowing that there is support during and after climate change disasters.

### Clarity

On this climate risk and others, a key question is whether L&D funds are channelled through governments, insurance companies, private sector or philanthropic organisations. With most of these funding mechanisms, there is a risk of focus on large-scale, top-down project interventions which may be less appropriate for cases less resilient to shocks and stresses [17]. Consequently, vulnerable and marginalised communities and migrants may not directly benefit from such infrastructure interventions [32]. To address this, clear rules and regulations on how to access this fund and when and what will be funded are needed. This sounds straightforward but has plagued past financial mechanisms and continues to stymie the discussions of the UNFCCC Transitional Committee. Indeed, Bergsvik et al. [56] have examined self-reporting sources by high-, middle- and low-income countries on climate finance and concluded that transparency does not promote clarity as there is still lack of multilateral agreed protocols and processes for climate finance. A major complaint about other funds has been: 1, the complexity of applying for the fund; 2, the types of projects funded; and 3, who can apply for the fund in the first place [32]. If the funds are to go to those most vulnerable, clarity on both contribution and attribution is essential.

### Community-driven

A real understanding of where the finance is coming from and who should be distributing these funds is needed. Climate adaption finance has historically allocated minimal resources towards community-led actions including the empowerment of the local community [57]. Similarly, UN funds can only be applied for at the national level. This approach to finance does not work for the L&D. The L&D fund needs a governance structure that not only champions local knowledge and solutions but also promotes their agencies in accessing and allocating funds towards local organisations that can implement and maintain climate adaptation solutions. To do so, a clearly defined, inclusive and accessible process to apply for and receive funding is needed, including bridging the knowledge gap, regulations, and the institutional requirements to access funds which are often incompatible with the institutional capacities and needs of local institutions [58]. For instance, loans are often the preferred instrument for climate adaptation funds as opposed to grant despite being unsuitable for some local organisations who are often challenged when it comes to credit worthiness [59,60]. Creating spaces for civil society groups and other community governance structures who are already working towards climate resilience to participate and co-design financial tools and implementation mechanisms would promote efficient and faster resources allocation. Further, empowering local communities in their own projects will lead to more effective and sustainable solutions.

### Corruption-free

The distribution of any funds needs to be corruption-free. Transparency and accountability for any fund are paramount in preventing misuse of funds. This goes back to point 3 (community-driven) on who can apply for such a fund, and then who distributes it. Establishing robust mechanisms for monitoring and reporting on fund utilisation can help maintain public trust and prevent misuse of resources.

## Conclusion

If these four principles of consistency, clarity, corruption-free and community-driven are carefully considered when designing and operationalising an L&D fund, the pitfalls of past mechanisms could be avoided.

The design of a new financial mechanism is a complex task. Incorporating these principles into the design and operation of the L&D fund can help address the frustrations and challenges associated with climate funding. It can also lead to more effective and equitable climate action, ultimately contributing to the global effort to combat climate change, while at the same time supporting communities affected by its impacts. These principles align with the goals of transparency, accountability and inclusivity in climate finance, which are crucial for achieving the objectives set out in the Paris Agreement. Ultimately, the fund will need to be reactive to climate events and predictive in understanding affected communities’ needs so that finance allocated is not just a band-aid solution but addresses the root causes of the vulnerabilities.

## Data Availability

Data sharing not applicable to this article as no datasets were generated or analysed during the current study.
